# Pelvic collection drainage by Heald anal stent

**DOI:** 10.1308/003588412X13373405385214d

**Published:** 2012-07

**Authors:** EJ Cook, BJ Moran, RJ Heald, GF Nash

**Affiliations:** ^1^Department of General Surgery,Poole General Hospital, Poole,UK; ^2^Department of General Surgery,North Hampshire Hospital, Basingstoke,UK

## BACKGROUND

The use of the Heald anal stent has previously been described in the successful therapeutic decompression of the rectum following a leaking ileorectal anastomosis^1^. The novel technique of using the Heald stent to drain a pelvic collection following rectal cancer surgery is presented. 

## TECHNIQUE

**Figure 1.**
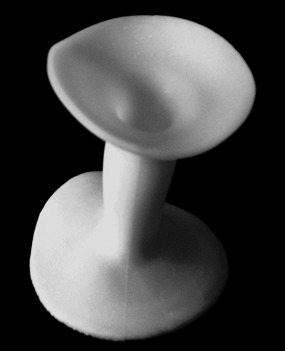
The hollow silicone elastomer Heald anal stent is flanged at both ends to prevent dislodgement in the anus.

The Heald anal stent (Fig 1) can be used to drain pelvic collections on the ward after any surgery that leaves a short rectal stump. The stent is inserted through the rectal cross-staples after the instillation of local anaesthetic gel. After several days, once drainage is complete, the stent is removed painlessly.

**Figure 2.**
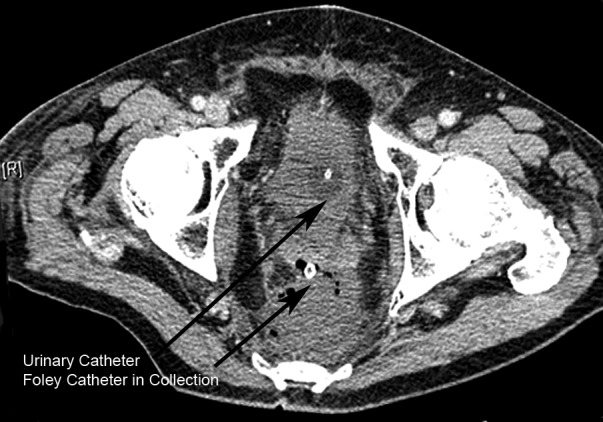
Computed tomography demonstrating pelvic fluid and gas collection incompletely drained by a rectal foley catheter

**Figure 3.**
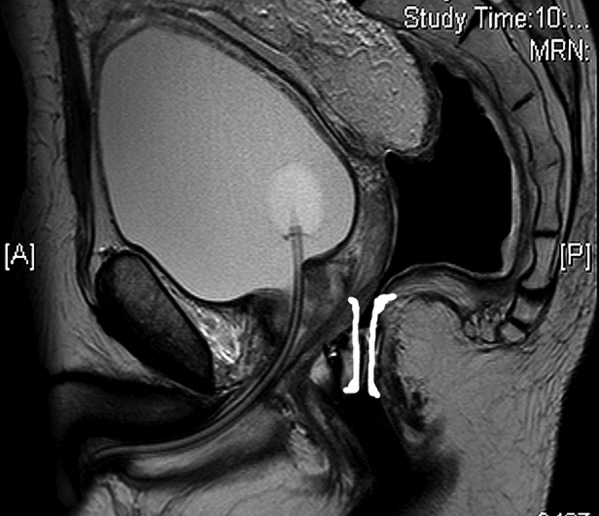
Sagittal magnetic resonance imaging of pelvis five days after the insertion of the Heald stent (highlighted) showing urinary catheter in the bladder and complete drainage of the presacral collection, now seen as air

We have used this technique successfully on patients with pelvic collections (Fig 2) who have failed foley catheter drainage. The stent may be left for a few days until drainage is complete. (pelvic magnetic resonance imaging may be used to confirm this [Fig 3].) The stent can then be removed on the ward.

## DISCUSSION

Pelvic sepsis is a common complication after colorectal surgery such as Hartmann’s operation. The risk is increased following neoadjuvant chemoradiotherapy, particularly in the presence of a suture or staple line.^2^ Foley catheters may be used to decompress pelvic collections but become blocked frequently. Being shorter and having a wider lumen, the Heald stent provides more effective drainage and is easy to irrigate if necessary. It has been previously demonstrated to be an alternative, albeit not certain, method of avoiding a defunctioning stoma in low rectal anastomoses.^3^ We recommend this technique as a possible method to allow free rectal drainage of a pelvic collection.
